# Fast Reconstruction of Compact Context-Specific Metabolic Network Models

**DOI:** 10.1371/journal.pcbi.1003424

**Published:** 2014-01-16

**Authors:** Nikos Vlassis, Maria Pires Pacheco, Thomas Sauter

**Affiliations:** 1 Luxembourg Centre for Systems Biomedicine, University of Luxembourg, Luxembourg City, Luxembourg; 2 Life Sciences Research Unit, University of Luxembourg, Luxembourg City, Luxembourg; The Centre for Research and Technology, Hellas, Greece

## Abstract

Systemic approaches to the study of a biological cell or tissue rely increasingly on the use of context-specific metabolic network models. The reconstruction of such a model from high-throughput data can routinely involve large numbers of tests under different conditions and extensive parameter tuning, which calls for fast algorithms. We present fastcore, a generic algorithm for reconstructing context-specific metabolic network models from global genome-wide metabolic network models such as Recon X. fastcore takes as input a core set of reactions that are known to be active in the context of interest (e.g., cell or tissue), and it searches for a flux consistent subnetwork of the global network that contains all reactions from the core set and a minimal set of additional reactions. Our key observation is that a minimal consistent reconstruction can be defined via a set of sparse modes of the global network, and fastcore iteratively computes such a set via a series of linear programs. Experiments on liver data demonstrate speedups of several orders of magnitude, and significantly more compact reconstructions, over a rival method. Given its simplicity and its excellent performance, fastcore can form the backbone of many future metabolic network reconstruction algorithms.

## Author Summary

Metabolism comprises all life-sustaining biochemical processes. It plays an essential role in various aspects of biology, including the development and progression of many diseases. As the metabolism of a living cell involves several thousands of small molecules and their conversion, a full analysis of such a metabolic network is only feasible using computational approaches. In addition, metabolism differs significantly from cell to cell and over different contexts. Therefore, the efficient generation of context-specific mathematical models is of high interest. We present fastcore, a fast algorithm for the reconstruction of compact context-specific metabolic network models. The algorithm takes as input a global metabolic model and a set of reactions that are known to be active in a given context, and it produces a context-specific model. fastcore is significantly faster than other algorithms, typically obtaining a genome-wide reconstruction in a few seconds. High-throughput model building will soon become a common procedure for the integration and analysis of omics data, and we foresee many future applications of fastcore in disease and patient specific metabolic modeling.

This is a *PLOS Computational Biology* Methods article.

## Introduction

Cell metabolism is known to play a key role in the pathogenesis of various diseases [1] such as Parkinson's disease [2] and cancer [3]. The study of human metabolism has been greatly advanced by the development of computational models of metabolism, such as Recon 1 [4], the Edinburgh human metabolic network [5], and Recon 2 [6]. These are genome-scale metabolic network models that have been reconstructed by combining various sources of ‘omics’ and literature data, and they involve a large set of biochemical reactions that can be active in different contexts, e.g., different cell types or tissues [7].

To maximize the predictive power of a metabolic model when conditioning on a specific context, for instance the energy metabolism of a neuron or the metabolism of liver, recent efforts go into the development of *context-specific* metabolic models [8]–[13]. These are network models that are derived from global models like Recon 1, but they only contain a subset of reactions, namely, those reactions that are active in the given context. Such context-specific metabolic models are known to exhibit superior explanatory and predictive power than their global counterparts [10], [14], [15].

Most algorithms for context-specific metabolic network reconstruction (see ‘Related work’ section for a short overview) first identify a relevant subset of reactions according to some ‘omics’ information (typically expression data and bibliomics), and then search for a subnetwork of the global network that satisfies some mathematical requirements and contains all (or most of) these reactions [8], [10], [13], [16]–[18]. The mathematical requirements are typically imposed via flux balance analysis, which characterizes the steady-state distribution of fluxes in a metabolic network via linear constraints that are derived from the stoichiometry of the network and physical conservation laws [19]–[23]. The search problem may target the optimization of a specific functionality of the model (e.g., biomass production) or some other objective [24], and it may involve repeated tests under different conditions and parameter tuning [8], [14], [25], [26]. The latter calls for fast algorithms.

We present fastcore, a generic algorithm for context-specific metabolic network reconstruction. fastcore takes as input a core set of reactions that are supported by strong evidence to be active in the context of interest. Then it searches for a *flux consistent* subnetwork of the global network that contains all reactions from the core set and a minimal set of additional reactions. Flux consistency implies that each reaction of the network is active (i.e., has nonzero flux) in at least one feasible flux distribution [19], [27]. An attractive feature of fastcore is its generality: As it only relies on a preselected set of reactions and a simple mathematical objective (flux consistency), it can be applied in different contexts and it allows the integration of different pieces of evidence (‘multi-omics’) into a single model.

Computing a minimal consistent reconstruction from a subset of reactions of a global network is, however, an NP-hard problem [27], and hence some approximation is in order. Our key observation is that a minimal consistent reconstruction can be defined via a set of *sparse* modes of the global network, and fastcore is designed to compute a minimal such set. Every iteration of the algorithm computes a new sparse mode via two linear programs that aim at maximizing the support of the mode inside the core set while minimizing that quantity outside the core set. fastcore's search strategy is in marked contrast to related approaches, in which the search for a minimal consistent reconstruction involves, for instance, incremental network pruning [10]. fastcore is simple, devoid of free parameters, and its performance is excellent in practice: As we demonstrate on experiments with liver data, fastcore is several orders of magnitude faster, and produces much more compact reconstructions, than the main competing algorithm MBA [10].

## Methods

### Background

A metabolic network of *m* metabolites and *n* reactions is represented by an *m*×*n stoichiometric* matrix *S*, where each entry *S_ij_* contains the stoichiometric coefficient of metabolite *i* in reaction *j*. A *flux* vector 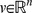 is a tuple of reaction rates, 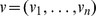, where 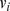 is the rate of reaction *i* in the network. Reactions are grouped into *reversible* ones (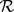) and *irreversible* ones (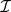). For a reaction 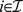 it holds that 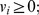 this and other imposed flux bounds, e.g., lower and upper bounds per reaction, are collectively denoted by 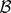 (which defines a convex set). A flux vector is called *feasible* or a *mode* if it satisfies a set of steady-state mass-balance constraints that can be compactly expressed as:
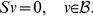
(1)An *elementary* mode is a feasible flux vector 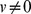 with minimal support, that is, there is no other feasible flux vector 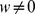 with 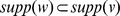, where 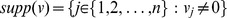 is the support (i.e., the set of nonzero entries) of 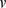 [19], [22]. A reaction *i* is called *blocked* if it cannot be active under any mode, that is, there exists no mode 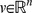 such that 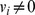 (in practice 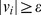, for some small positive threshold *ε*). A metabolic network model that contains no blocked reactions is called *(flux) consistent* [19], [27].

### Network consistency testing

Given a metabolic network model with stoichiometric matrix *S*, a problem of interest is to test whether the network is consistent or not. Additionally, if the network is inconsistent, it would be desirable to have a method that detects all blocked reactions.

**Figure 1:**
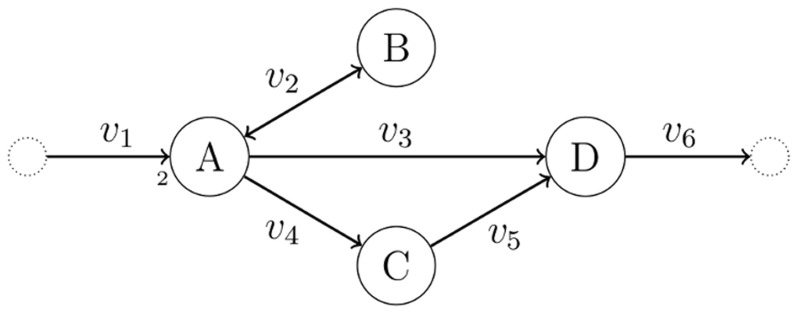
A metabolic network with one blocked reaction (A↔B). Note that A appears with stoichiometric coefficient 2 in the boundary reaction →2A.

It has been suggested that network consistency can be detected by a single linear program (LP) [27]. The idea is to first convert each reversible reaction into two irreversible reactions (and define a reversible flux as the difference of two irreversible fluxes), and then test if the minimum feasible flux on the new set 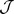 of irreversible-only reactions is strictly positive (in practice, at least *ε*). This is equivalent to testing if the following LP is feasible:
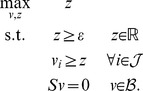
(LP - 2)This test of consistency, however, can produce spurious solutions. In Figure 1 we show a toy metabolic network comprising four metabolites (A,B,C,D) and six reactions annotated with corresponding fluxes 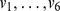. Fluxes are bounded as 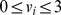 for 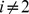, and 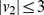. All stoichiometric coefficients are equal to one, except for the reaction →2A. The only reversible reaction is A↔B, which is a dead-end reaction and therefore blocked, whereas all other reactions are irreversible and unblocked. After converting A↔B to a pair of irreversible reactions, LP-2 achieves optimal value 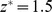, which implies (wrongly) that the network is consistent. The test here fails because the two irreversible copies of A↔B have equal flux at the solution, thereby nullifying the actual net flux of A↔B.

A straightforward solution to the problem would involve iterating through all reactions, computing the maximum and minimum feasible flux of each reaction via an LP that satisfies the constraints in (1). Reactions with minimum and maximum flux zero would then be blocked. This is the idea behind the FVA (Flux Variability Analysis) algorithm and the *reduceModel* function of the COBRA toolbox [28], [29]. However, iterating through all reactions can be inefficient. A faster variant is fastFVA [30], which achieves acceleration over FVA via LP warm-starts. Another fast algorithm is CMC (CheckModelConsistency) [10], which involves a series of LPs, where each LP maximizes the sum of fluxes over a subset 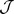 of reactions:
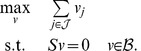
(LP - 3)The set 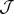 is initialized by 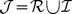 (all reactions in the network), and it is updated after each run of LP-3 so that it contains the reactions whose consistency has not been established yet. When 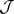 cannot be reduced any further, we can reverse the signs of the columns of *S* corresponding to the reversible reactions in 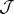 and resume the iterations. Eventually, all remaining reactions may have to be tested one by one for consistency, as in FVA. Such an iterative scheme is complete, in the sense that it will always report consistency if the network is consistent, and if not, it will reveal the set of blocked reactions. However, as we will clarify in the next section, LP-3 is not optimizing the ‘correct’ function, which may result in unnecessarily many iterations. For example, when applied to the network of Figure 1, LP-3 will pick up the elementary mode that corresponds to the pathway A→C→D (because this pathway achieves maximum sum of fluxes 
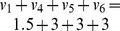
), and it will set 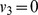. To establish the consistency of the reaction A→D, an additional run of LP-3 would be needed, where the set 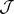 would only involve the reactions A↔B and A→D. Hence, an iterative algorithm like CMC that relies on LP-3 would need two iterations to detect the consistent part of this network. However, one LP suffices to detect the consistent subnetwork in this example, as we explain in the next section. In more general problems involving larger and more realistic networks, CMC may involve unnecessarily many iterations, as we demonstrate in the experiments.

### Fast consistency testing

In most problems of interest there will be no single mode that renders the whole network consistent, and an iterative algorithm like the one described in the previous section must be used. For performance reasons it would therefore be desirable to be able to establish the consistency of as many reactions as possible in each iteration of the algorithm.

Since consistency implies nonzero fluxes, it is sufficient to optimize a function that just ‘pushes’ all fluxes away from zero. Formally, this amounts to searching for modes 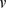 whose *cardinality*—denoted by *card(v)* and defined as *card(v) = #supp(v)*, i.e., the number of nonzero entries of 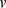
—is as large as possible. Directly maximizing *card(v)* is, however, not straightforward, for the following reasons: First, the *card* function is quasiconcave only for 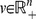 (the nonnegative orthant), and it is nonconvex for general 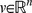 [31]. Second, even if we restrict attention to nonnegative fluxes in each iteration (which we can do without loss of generality by flipping the signs of the corresponding columns of *S*), it is not obvious how to efficiently maximize the quasiconcave *card(v)*. Third, in practice consistency implies fluxes that are *ε*-distant from zero, in which case some adaptation of the *card* function is in order.

Here we propose an approach to approximately maximize *card(v)* over a nonnegative flux subspace indexed by a set of reactions 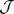. First note that the cardinality function can be expressed as
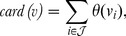
(4)where 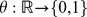 is a step function:
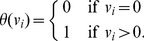
(5)The key idea is to approximate the function *θ* by a concave function that is the minimum of a linear function and a constant function:
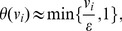
(6)where *ε* is the flux threshold. The problem of approximately maximizing *card(v)* can then be cast as an LP: We introduce an auxiliary variable 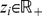 for each flux variable 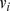, for 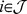, and take epigraphs [31], in which case maximizing *card(v)*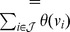 can be expressed as
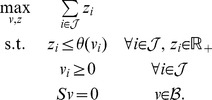
Using (6) and assuming constant *ε*, this simplifies to
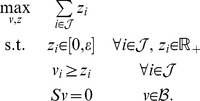
(LP-7)Note that LP-7 tries to maximize the number of feasible fluxes in 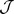 whose value is at least *ε* (contrast this with LP-2).

Returning to the network of Figure 1, if 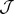 comprises all network reactions, then note that the flux vector 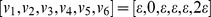 is an optimal solution of LP-7. Hence, a single run of the latter can detect all unblocked reactions of that network. More generally, a single run of LP-7 on an arbitrary subset 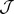 of a given network will typically detect all unblocked *irreversible* reactions of 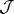. The intuition is that LP-7 prefers flux ‘splitting’ over flux ‘concentrating’ in order to maximize the number of participating reactions in the solution, which, in the case of irreversible reactions, corresponds to flux cardinality maximization.

By construction, the above approximation of the cardinality function applies only to nonnegative fluxes. In order to deal with reversible reactions that can also take negative fluxes, we can embed LP-7 in an iterative algorithm (as in the previous section), in which reversible reactions are first considered for positive flux via LP-7, and then they are considered for negative flux. The latter is possible by flipping the signs of the columns of the stoichiometric matrix that correspond to the reversible reactions under testing, in which case the fluxes of the transformed model are again all nonnegative, and the above approximation of the cardinality function can be used. This gives rise to an algorithm for detecting the consistent part of a network that we call fastcc (for fast consistency check). Since fastcc is just a variant of fastcore, we defer its detailed description until the next section.

Independently to this work, a similar approach to network consistency testing was recently proposed, called OnePrune [32]. OnePrune first converts each reversible reaction into two irreversible reactions, forming an augmented set 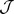 of irreversible-only reactions (as in LP-2 above), and then it employs an LP that coincides with LP-7 for the above choice of 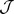 and *ε* = 1. However, such an approach is prone to the same drawback as LP-2, namely, that the two irreversible copies of a blocked reaction can carry equal positive flux at the solution of LP-7 due to the presence of cycles introduced by the transformation. The authors acknowledge this problem but they do not fully resolve it. In our case, we avoid this problem by working with the original reactions and a series of LPs with appropriate sign flips of the stoichiometric matrix, thereby guaranteeing the completeness of the algorithm.

### Context-specific network reconstruction

The reconstruction problem involves computing a minimal consistent network from a global network and a ‘core’ set of reactions that are known to be active in a given context. Formally, given (i) a *consistent* global network 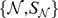 with reaction set 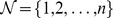 and stoichiometric matrix 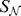, and (ii) a set 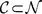, the problem is to find the smallest set 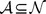 such that 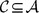 and the subnetwork 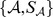 induced by the reaction set 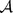 is consistent. (By 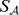 we denote the submatrix of 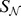 that contains only the columns indexed by 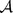.) This problem is known to be NP-complete [27], suggesting that a practical solution should entail some approximation. (We note that Acuña et al. [27] prove NP-completeness of this problem by noting that a special case involves 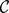 being the empty set, in which case the problem comes down to finding the smallest elementary mode of the global network, which, as the authors show, is NP-complete. However, this leaves open the case of a nonempty core set 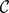, since a solution to the minimal reconstruction problem need not constitute an elementary mode. We conjecture that the problem remains NP-hard when 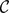 is nonempty, but we are not pursuing this question here.)

Our approach hinges on the observation that a consistent induced subnetwork of the global network can be defined via a set of modes of the latter:

**Theorem 1.** *Let *
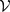
* be a set of modes of the global network *
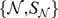
*, and let *
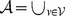
*supp(v) be the union of the supports of these modes. The induced subnetwork *
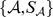
* is consistent.*

*Proof.* For each 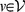, let 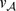 be the ‘truncated’ 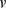 after dropping all dimensions not indexed by 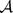. Clearly, 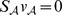, therefore each 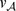 is a mode in the reduced model 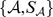. By construction of 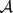, each reaction in 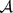 is in the support of some 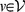, and hence also in the support of some mode 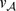 of the reduced model. 

This simple result allows one to cast the reconstruction problem as a search problem over sets of modes of the global network:
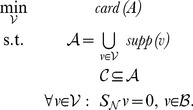
(NLP-8)Note that this optimization problem involves searching for a set 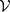 of modes of 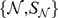, such that the union of the support of these modes (the set 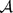) is a minimal-cardinality set that contains the core set 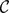. In order to practically make use of this theorem, one has to define a search strategy over modes. Next we discuss two possibilities. The first gives rise to an exact algorithm, but this algorithm does not scale to large networks. The second is a scalable greedy approach that gives rise to fastcore.

#### Exact reconstruction via mixed integer linear programming

Note that, without loss of generality, in NLP-8 we can restrict the search for 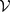 over all *elementary modes* of the global network, since the union of their supports covers the whole set 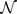. As we show next, if all elementary modes are available, NLP-8 can be cast as a mixed integer linear program (MILP) and solved exactly. This MILP is defined as follows. Let *r* be the number of elementary modes, and 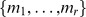 be a set of length-*n* binary vectors, where each vector *m_j_* captures the support of elementary mode *j* (so, its *i*th entry is 1 if reaction *i* is included in elementary mode *j*, and 0 otherwise). Also, let 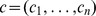 be a length-*n* binary vector with 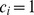 if reaction *i* is included in the core set 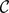, and 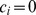 otherwise. The decision variables of the MILP are a length-*n* binary vector 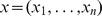 and a length-*r* real vector 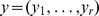. At an optimal solution of the MILP, the set 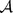 is defined as 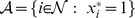.

**Theorem 2.** *When all elementary modes are available, the following MILP-9 solves NLP-*8 *exactly.*
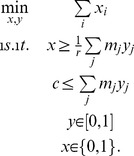
(MILP-9)

*Proof.* By definition, 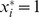 implies that reaction *i* will be included in the reconstruction 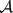, hence the objective minimizes the cardinality of 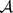. The sum 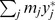 is a vector whose support is the union of the supports of all selected elementary modes at the solution, where an elementary mode *j* is selected when 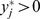. The first constraint 
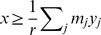
 therefore imposes that the set 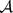 must contain the union of the supports of the selected elementary modes at the solution. (The factor 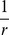 ensures that 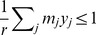). Since superfluous reactions are removed by the minimization of 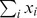 in the objective, the above implies that 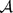 is precisely the union of the supports of the selected elementary modes at the solution. The second constraint 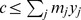 imposes that the core set must be included in the union of the supports of the selected elementary modes at the solution, and hence the core set must be included in 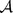. Therefore, all constraints of NLP-8 are satisfied at the optimal solution of MILP-9, and since the two programs minimize the same objective, an optimal solution of MILP-9 must be an optimal solution of NLP-8.

Note, however, that MILP-9 does not scale to large networks, for the following reasons: First, it requires computing all elementary modes of the global network, which can be a very large number [22]. Second, the binary decision variables 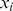 index all reactions of the global network, and therefore MILP-9 needs to search over a binary hypercube of dimension *n*, which can be prohibitive for large *n*. Nonetheless, it is reassuring to know that an exact solution to the context-specific network reconstruction problem is possible, albeit with high complexity. Next we describe fastcore, an approximate greedy algorithm that scales much better to large networks, and we compare it to MILP-9 in the Results section.

#### Greedy approximation and the fastcore algorithm

An alternative search strategy for computing 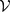 in NLP-8 is a greedy approach, reminiscent of greedy heuristics for the related *set covering problem* [33]. This is the idea behind the proposed fastcore algorithm: We build up the set 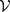 in a greedy fashion, by computing in each iteration a new mode of the global network. Further, as a means to approximately minimize *card(A)*, each added mode is constrained to have *sparse* support outside 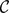. This is implemented via *L*_1_-norm minimization, which is a standard approach to computing sparse solutions to (convex) optimization problems [31], [34].

The overall fastcore algorithm is shown in Box 1. The algorithm maintains a set 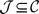 that is initialized with the irreversible reactions in 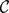, and a ‘penalty’ set 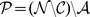 that contains all reactions outside 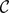 that have not been added yet to the set 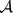. Each iteration adds to the set 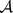 the support of a mode that is dense in 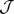 (i.e., contains as many nonzero fluxes in 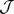 as possible) and sparse in 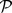 (i.e., contains as many zero fluxes in 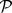 as possible), computed by the function FindSparseMode (Box 2). This function first applies an LP-7 to compute an active subset 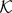 of 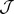, and then it applies the following *L*_1_-norm minimization LP constrained by the set 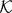:
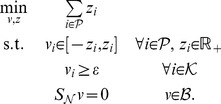
(LP-10)The LP-10 minimizes 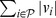, the *L*_1_ norm of fluxes in the penalty set 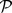 (expressed via epigraphs), subject to a minimum flux constraint on the set 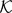. However, some care is needed to preempt false negative solutions arising from the minimization of *L*_1_ norm in LP-10. For example, suppose in the network of Figure 1 that the global network comprises all reactions except A↔B, and 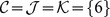 and 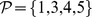. In this case, LP-3 could settle to a solution 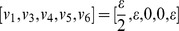. The flux 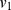, being below *ε*, would be treated as zero by FindSparseMode, in which case the reaction →2A would be erroneously excluded from the reconstruction. A simple way to avoid this is to use a scaled version of *ε* (we used 10^5^*ε*) in the second constraint of LP-10, with an equal scaling of all flux bounds in 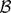.

**Box 1. The fastcore Algorithm for Context-Specific Metabolic Network Reconstruction**

Input: A consistent metabolic network model 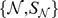 and a reaction set 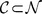.

Output: A consistent induced subnetwork 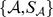 of 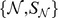 such that 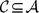.

1.  **function** fastcore(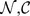)
  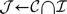, 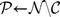
  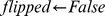, 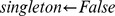
  
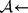
FINDSPARSEMODE
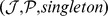
  
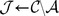
6.   **while** 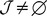
   
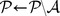
   
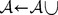
FINDSPARSEMODE
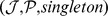
9.    **if** 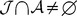
    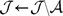, 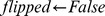
11.   **else**
12.     **if** 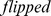
     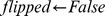, 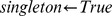
14.     **else**
     
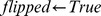
16.      **if** *singleton*
      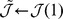 (the first element of 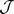)
18.      **else**
      
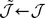
20.     **end if**
21.     **for each** 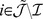
22.      flip the sign of the *i*'th column of 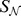 and
23.      swap the upper and lower bounds of 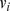
24.     **end for**
25.     **end if**
26.    **end if**
27.   **end while**
28.   **return** 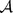
29.  **end function**

**Box 2. The FindSparseMode Function**

Input: A set 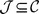, a penalty set 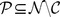, and the *singleton* flag.

Output: The support of a mode that is dense in 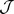 and sparse in 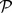.

1.  **function** FindSparseMode(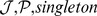)
2.   **if** 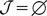
3.    **return** 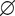
4.   **end if**
5.   **if** *singleton*
   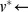 LP-7 on set 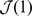
7.   **else**
   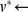 LP-7 on set 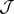
9.   **end if**
  
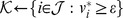
11.   **if** 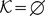
12.    **return** 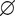
13.    **end if**
   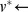 LP-10on sets 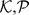
15.    **return** 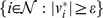
16.  **end function**

The fastcore algorithm first goes through the 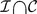 reactions (step 2), and then through the 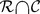 ones (and eventually through each individual reversible reaction in the core set; when *singleton* = *True*). The *flipped* variable ensures that a reversible reaction is tested in both the forward and negative direction. The algorithm terminates when all reactions in 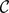 have been added to 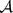, which is guaranteed since in the main loop the set 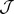 never expands (step 10) and the global network is consistent. Note that fastcore has no free parameters besides the flux threshold *ε*.

The fastcc algorithm for detecting the consistent part of an input network (see previous section) can be viewed as a variant of fastcore 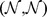 in which the steps 10–14 of FindSparseMode are omitted (and there is no 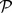 set). It is easy to verify that fastcc is complete, in the sense that it will always report consistency if the network is consistent, and if not, it will reveal the set of blocked reactions.

### Related work

Several algorithms have been published in the last years for extracting condition-specific models from generic genome-wide models like Recon 1. Among them, mCADRE [26], INIT [13], iMAT [35], MBA [10] and GIMME [8] are the most commonly used (see Table 1 for an overview). Here we provide a short outline of the different algorithms, and refer to [24] for a more extensive overview. For GIMME, iMAT, and MBA, we briefly discuss some notable differences to fastcore.

**Table 1:** Summary of the main characteristics of GIMME [8], MBA [10], iMAT [35], mCADRE [26], INIT [13], and fastcore (this paper) reconstruction algorithms.

	GIMME	MBA	iMAT	mCADRE	INIT	FASTCORE
Optimization	LP	MILP	MILP	MILP	MILP	LP
Computational cost	low	high	high	high	high	low
Function required	yes	no	no	yes	yes	no
Omics required	yes	optional	yes	yes	yes	no
Code available	yes	yes	yes	yes	no	yes

GIMME [8] takes as input microarray data and a biological function to optimize for, such as biomass production. GIMME starts by removing reactions with associated expression levels below a user-defined threshold, and then it optimizes for the specified biological function using linear programming. In case the pruning steps compromise the input biological function, GIMME reintroduces some previously removed reactions that are in minimal disagreement with the expression data. Since GIMME has not been designed to include all core reactions in the solution (as fastcore does), the reconstructions obtained by GIMME and fastcore can differ significantly: Running the *createTissueSpecific* function of the COBRA toolbox on a set of liver core reactions (see ‘Results’ section) treating them as expressed reactions (and adding a biomass reaction [26] and a sink reaction for glycogen to be used as optimization function), only about 50% of the core reactions of the GIMME model were consistent at the solution. A fairer comparison would require adapting fastcore to explicitly deal with omics data, which is outside the scope of the current work.

iMAT [35] was originally designed for the integration of transcriptomic data. iMAT optimizes for the consistency between the experimental data and the activity state of the model reactions. iMAT tries to include modes composed of reactions associated to genes with high expression value, and therefore a threshold needs to be chosen to segregate between low, medium, and highly expressed genes. The computational demands of iMAT are high due to the repeated use of mixed integer linear programming. As with GIMME, direct comparison of iMAT to fastcore is problematic. Nevertheless, we applied iMAT (own implementation) on the liver problem (see ‘Results’ section), by setting the liver core reactions to RH (reaction high) and all non-core reactions to RL (reaction low). iMAT determined 549 core reactions as active, while 182 and 338 reactions were classified as undetermined and inactive, respectively. This means that about 50% of the core reactions were lost during iMAT model building. As with GIMME, this demonstrates the difficulty of directly comparing fastcore to algorithms that optimize different objectives.

mCADRE [26] is similar to MBA, except that the pruning order is not random, but it depends on the tissue-specific expression evidence and weighted connectivity to other reactions of the network. Reactions that are associated to genes that are never tagged as expressed and which are not connected to reactions associated to highly expressed genes are first evaluated in the pruning step. Reactions are effectively removed if the removal does not impair core reactions and metabolic functions to carry a flux (mCADRE removes core reactions if the core/non-core reaction ratio is below a user-given threshold). mCADRE uses mixed integer linear programming and therefore it does not scale up to large networks (but it is in general faster than MBA).

INIT [13] uses data retrieved from public databases in order to assess the presence of a certain reaction-respective metabolites in the cell type of interest. INIT uses mixed integer linear programming to build a model in which all reactions can carry a flux. Contrary to other algorithms, INIT does not rely on the assumption of a steady state, but it allows small net accumulation of all metabolites of the model.

The closest algorithm to fastcore is the MBA algorithm of Jerby et al. [10]. MBA takes as input two core sets of reactions, and it searches for a consistent network that contains all reactions from the first set, a maximum number of reactions from the second set (for a given tradeoff), and a minimal number of reactions from the global network. (fastcore can be easily adapted to work with multiple core sets, by introducing a set of weights that reflect the confidence of each reaction to be active in the given context, and adding appropriate regularization terms in the objective functions of LP-7 and LP-10 that capture the given tradeoff. We will address this variant in future work.) Both fastcore and MBA involve a search for a minimal consistent subnetwork, however the search strategy of fastcore is very different to MBA: Whereas fastcore iteratively expands the active set 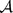 starting with 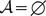, MBA starts with 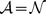 and iteratively prunes the set 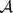 by checking whether the removal of each individual reaction (selected in random order) compromises network consistency. As the pruning order affects the output model, this step of MBA is repeated multiple times. MBA builds a final model by adding one by one non-core reactions with the highest presence rate over all pruning runs, and it stops when a consistent final model is obtained. Due to the multiple pruning runs, MBA has very high computational demands. Consistency testing in MBA is carried out with the CMC algorithm that is based on LP-3, as explained earlier. Hence, fastcore's search strategy differs to MBA in two key aspects: First, consistency testing in fastcore involves the maximization of flux cardinality (LP-7) instead of sum of fluxes (LP-3), which results in fewer LP iterations. Second, the search for compact solutions in fastcore involves *L*_1_-norm minimization instead of pruning. The advantage of the former is that it can be encoded by a single LP, resulting in significant overall speedups (see ‘Results’ section).

## Results

**Figure 2:**
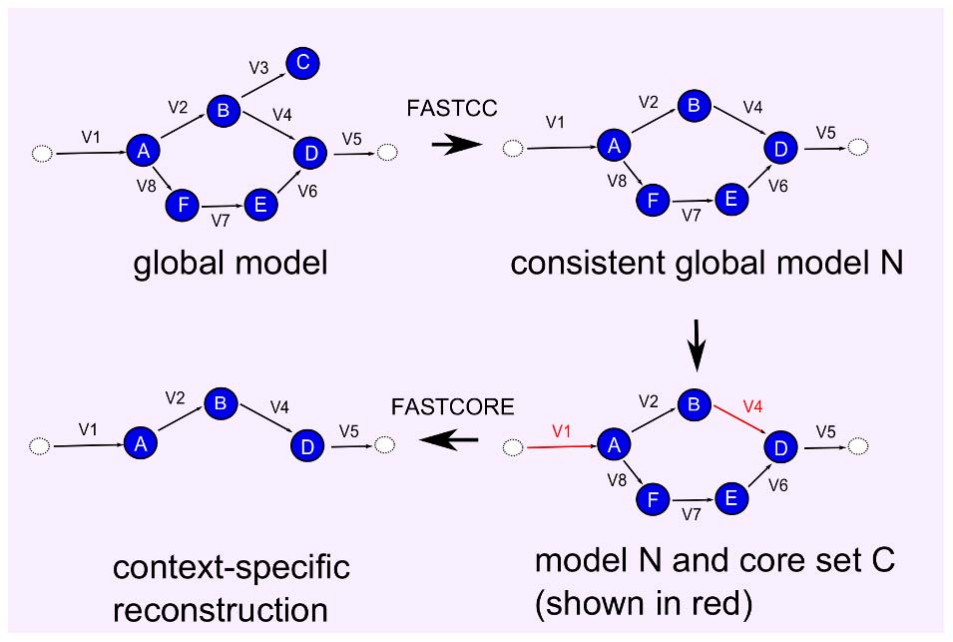
Flowchart of the overall pipeline for generating consistent context-specific models.

Generic metabolic reconstructions like Recon 2 are inconsistent models as they contain reactions that are not able to carry nonzero flux due to gaps in the network (see next section). The first step towards obtaining a consistent context-specific reconstruction is therefore to extract the consistent part of a global generic model. This can be achieved by fastcc or other similar methods (see ‘Network consistency testing’ section). The consistent global model serves then as input for the context-specific reconstruction with fastcore. In Figure 2 we show a flowchart of the overall pipeline.

We report results on two sets of problems, the first involving consistency verification of an input model, and the second involving the reconstruction of a context-specific model from an input model and a core set of reactions. The fastcore algorithm was implemented in the COBRA toolbox [29], using Matlab 2013a and the IBM CPLEX solver (version 12.5.0.0). Test runs were performed on a standard 1.8 GHz Intel Core i7 laptop with 4 GB RAM running Mac OS X 10.7.5. In all experiments we used flux threshold *ε* = 1e-4. The software is available from bio.uni.lu/systems_biology/software

### Consistency testing

In the first set of experiments we applied fastcc, the consistency testing variant of fastcore, for consistency verification of four input models, and compared it against the FastFVA algorithm of Gudmundsson and Thiele [30], and an own implementation (based on fastcc but with LP-3 replacing LP-7) of the CMC algorithm of Jerby et al. [10]. We also tested the FVA algorithm of the *reduceModel* function of the COBRA toolbox [29], and the MIRAGE algorithm of Vitkin and Shlomi [36], but we do not include them in the results as they performed worse than the reported ones. The input models were the following:

- c-Yeast (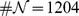), the consistent part of a yeast model [37].
- c-Ecoli (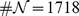), the consistent part of an *E. coli* model [25]. (Here we set to 1000 the upper bounds of all fluxes that were fixed to zero, and we multiplied all bounds by 1000 to avoid numerical issues.)
- c-Recon1 (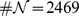), the consistent part of Recon 1 [4]. (Recon 1 was found to contain 1273 blocked reactions.)
- c-Recon2 (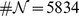), the consistent part of Recon 2 [6]. (Recon 2 was found to contain 1606 blocked reactions.)

The results are shown in Table 2. fastcc is faster and it uses much fewer LPs than the other two algorithms. We note that fastFVA is based on an optimized Matlab/C++ implementation with LP warm-starts, while fastcc is based on standard Matlab. These results confirm the appropriateness of flux cardinality (LP-7) as a metric for network consistency testing, in agreement with the theoretical analysis and the discussions above.

**Table 2:** Comparing fastcc to fastFVA [30] and CMC [10] on four input models.

	c-Yeast		c-Ecoli		c-Recon1		c-Recon2	
	#LPs	time*	#LPs	time	#LPs	time	#LPs	time
fastFVA	2408	3	3436	3	4938	9	11668	207
CMC	18	0.5	25	1	49	2	42	11
fastcc	7	0.1	2	0.2	9	0.4	19	5

in seconds.

### Reconstruction of a liver model

In the second set of experiments, we used the fastcore algorithm to reconstruct a liver specific metabolic network model from the consistent part of Recon 1 (c-Recon1, 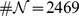), and we compared against an own implementation of the MBA algorithm of Jerby et al. [10]. We applied the two algorithms in two settings. The first setting involves the liver specific input reaction set of Jerby et al. [10], which is based on 779 ‘high’ core and 290 ‘medium’ core reactions (the latter set is supported by weaker biological evidence than the former). To allow a comparison with fastcore, we defined a single core set as the union of the high and medium core reaction sets, and we applied the two algorithms on this core set. The second setting uses the ‘strict’ liver model of Jerby et al. [10], which contains 1083 high core reactions and no medium core reactions, and therefore allows a direct comparison with fastcore.

The results for the two settings are shown in Table 3. We note that for MBA, the reported number of LPs and the runtime refer to a single pruning iteration of the algorithm, whereas the size of each reconstruction refers to the final model after 1000 pruning iterations. In both settings, fastcore is several orders of magnitude faster than MBA, achieving a full reconstruction of a liver specific model in about one second, using a much smaller number of LPs. As MBA employs a greedy pruning strategy for optimization, the number of LPs that it uses and its total runtime can be very high, as also indicated by Wang et al. [26] who reported runtime of a single pruning pass of MBA in the order of 10 hours on a 2.34 GHz CPU computer.

**Table 3:** Comparing fastcore to MBA [10] on liver model reconstruction from c-Recon1.

	liver core set ( 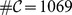 )				strict liver core set ( 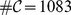 )			
	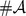	IR*	#LPs	time†	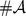	IR	#LPs	time
MBA	1826	1573	72279	7383	1887	1630	71546	6730
fastcore	1746	1546	20	1	1818	1627	20	1

number of intracellular reactions.

the reported time (in seconds), as well as the number of LPs, refer to a single pruning step of MBA, whereas 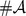 and IR refer to the full MBA.

The reconstructed models by fastcore are also more compact than those obtained by MBA, with a difference of 70–80 non-core reactions. For the standard liver model, 1687 out of the 1746 reactions (96%) of the fastcore reconstruction appear also in the MBA reconstruction, whereas for the strict liver model the common reactions are 1739 out of 1818 (95%). The two algorithms turned out to use alternative transporters to connect the core reactions: In the standard liver model, 46 out of 59 reactions that are present exclusively in the fastcore reconstruction are transporter reactions or other reactions which are not associated to a specific gene and thus are not sufficiently supported in the core set, whereas in MBA the corresponding numbers are 116 out of 139 reactions. (In Text S1 we provide more details on the reconstructions obtained by the two methods.) Note that both MBA and fastcore try to minimize the number of added non-core reactions in order to obtain a compact consistent model. The above difference in the number of added non-core reactions between MBA and fastcore is the result of the different optimization approaches taken by the two algorithms, and no biological relevance should be attributed to each reconstruction other than the one implied by the makeup of the core set. From this point of view, fastcore performs in general better than MBA, as it tends to add fewer unnecessary reactions.

**Figure 3:**
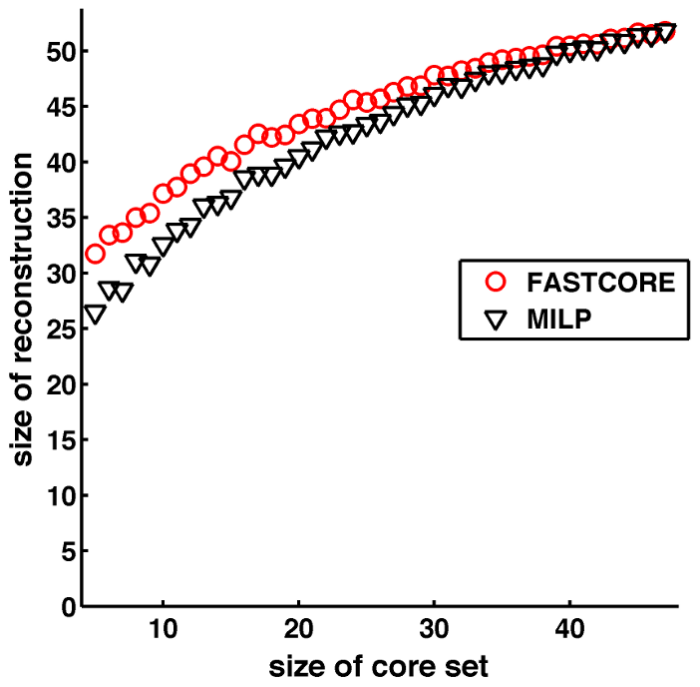
Comparing fastcore to an exact MILP solver on a small *E. coli* model [38]. Shown are mean values of sizes of reconstructed models (over 50 repetitions for each core set; standard deviations were small and are omitted to avoid clutter) as a function of the size of the core set. fastcore computes near-optimal reconstructions, which improve with the size of the core set.

We also compared fastcore's reconstructions to the exact solutions obtained from MILP-9, using core sets that are randomly generated from a consistent subset of *E. coli core* [38]. This is a small model with 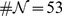 and 414 elementary modes (unfortunately, the dependence of the MILP-9 model on the number of elementary modes did not allow testing larger models). In Figure 3 we show the size of the reconstructed models (mean values) obtained with the MILP formulation vs. fastcore, as a function of the size of the core set. fastcore is capable of obtaining very good approximations to the optimal solutions, which improve with the size of the core set.

To evaluate fastcore's performance in correctly identifying liver reactions, we performed repeated random sub-sampling validation in which fastcore was used to reconstruct the liver metabolism based on a reduced, randomly selected ‘subcore’ set of 80% of the original core reactions. As in [10], we wanted to test whether fastcore is able to recover a significant number of the 20% left-out core reactions. To test for the enrichment of the left-out core reactions in the reconstructed model, we used a hypergeometric test, in which the total population is defined by all non-subcore reactions in the global network, the number of draws is defined as the number of non-subcore reactions included in the reconstruction, and the left-out core reactions are the ‘successes’. Under the null-hypothesis that there is no enrichment for the left-out core reactions when reconstructing the liver model based on the subcore set, we can compute a p-value for including at least the number of observed left-out core reactions in the reconstruction. We repeated this random sub-sampling procedure 500 times and computed the corresponding p-values. The median of these p-values was 0.0025, indicating the ability of fastcore to capture liver-specific reactions that were included in the original core set.

**Figure 4:**
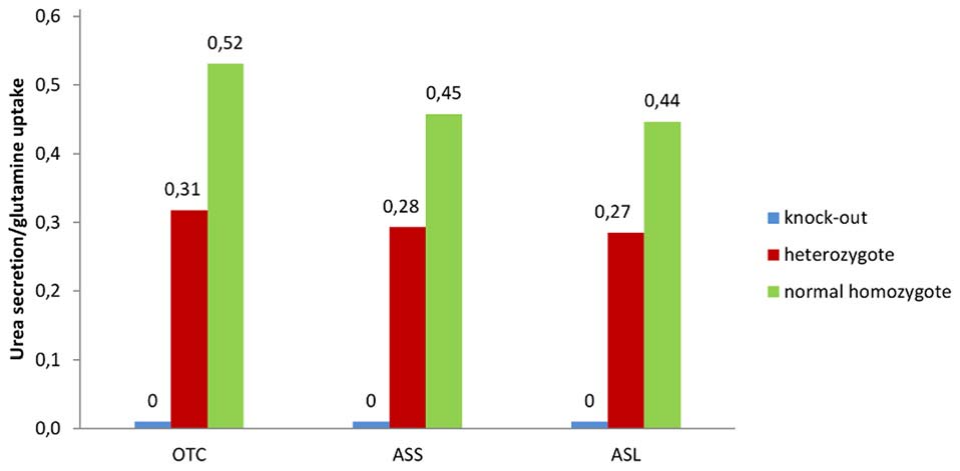
Mean urea/glutamine ratio in the extended liver model obtained by fastcore. Healthy (normal homozygote), partial (heterozygote) and full knock-out cases. See text for details.

As argued above, the reconstructions obtained by fastcore need not optimize for cellular functions other than the ones implied by the composition of the input core set, and it is an interesting research question how to modify fastcore so that it can explicitly capture functional requirements in its reconstructions. Nevertheless, it is of interest to test whether the current version of fastcore can produce reconstructions that *are* functionally relevant, perhaps for slight variations of the core set. To this end, as in [10], we checked whether the (standard) liver model reconstructed by fastcore can perform gluconeogenesis from glucogenic amino acids, glycerol, and lactate (altogether 21 metabolites). If not yet included, transporters from the extracellular medium to the cytosol were added to the model (glycerol, glutamate, glycine, glutamine, and serine). This was necessary as the transport reactions were not sufficiently supported in the core set. This ‘extended’ liver model was able to convert 17/21 metabolites (vs 12/21 metabolites of the non-extended model). The extended liver model was then used to simulate the liver disorders hyperammonemia and hyperglutamenia, which affect the capacity to metabolize dietary amino acids into urea [10]. Loss of function mutations of three enzyme-coding genes, argininosuccinate synthetase (ASS), argininosuccinate lyase (ASL), and ornithine transcarbamylase (OTC) were identified in patients suffering from these disorders. The rates of the reactions controlled by the three genes were fixed to 500, 250, or zero, to mimic the healthy homozygote (no mutation), heterozygote (loss of one allele), and the complete loss of function, respectively. To allow for a comparison with the experimental study of Lee et al. [39] where labeled 15N-glutamine was administrated to patients suffering from inborn errors affecting the three genes, we explicitly shut down the influx of other potential nitrogen sources in the liver model, thereby simulating only the uptake and metabolism of glutamine. By allowing the influx of only one nitrogen source, the fate of the latter could be determined exactly in the model. The ratio of urea secretion level over glutamine absorption was computed by sampling over the feasible space [21]. In accordance with the wet lab observations [39], the severity of the disorders, characterized by the mean urea over glutamine ratio, increased with the level of loss of function of the three genes ASS, ASL, and OTC (see Figure 4). Null patients showed no native production of urea. Overall, the ratios predicted by the fastcore model faithfully match the experimentally observed ones [39]. (The corresponding ratios reported by Jerby et al. when using the MBA algorithm [10] matched less well the experimental observations, probably because of the cross-feeding of nitrogen to urea from multiple nitrogen sources. By running the above procedure on the MBA model, we noticed that both models attained comparable urea/glutamine flux ratios.) To summarize, the above experiments demonstrate that, by an informed choice of the core set and influx bounds, fastcore can indeed give rise to functionally relevant models.

### Reconstruction of a murine macrophage model

We also used the fastcore algorithm to build a cell-type specific murine macrophage model from the consistent part of Recon1bio (comprising 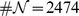 reactions). Recon1bio (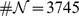) is a modified Recon 1 model that contains three extra reactions (biomass, NADPOX, and a sink reaction to balance the glycogenin self-glucosylation reaction) [15]. We used a core set comprising 300 (out of 382) proteomics derived Raw264.7 macrophage reactions, as described by Bordbar et al. [15]. (The remaining 82 reactions could not be added to the core set as they are situated in an inconsistent region of Recon 1 and therefore carry a permanent zero net flux.) For their macrophage reconstruction, Bordbar et al. used, among other methods, GIMMEp—a variant of the GIMME algorithm [8] that is similar to the MBA algorithm—and they obtained a network model containing 1026 intracellular reactions. Our main interest was to investigate whether fastcore can obtain a functional network that is at least as compact as the one obtained with GIMMEp. fastcore generated (in about one second and using 11 LPs) a consistent network model of 953 reactions, 831 of which are intracellular reactions. This is a much more compact model than the one obtained with GIMMEp.

## Discussion

fastcore is a generic algorithm for context-specific metabolic network reconstruction from genome-wide metabolic models, and it was motivated by requirements of fast computation and compactness of the output model.

The key advantage of having a fast reconstruction algorithm is that it permits the execution of multiple runs in order to optimize for extra parameters or test different core sets extracted from the input data [14], [26]. For example, when working with gene expression data, the definition of the core set may depend on the threshold used to segregate between high expression genes (core reactions) and low expression genes (non-core reactions) [8]. As the choice of threshold is rather arbitrary, a practical approach could involve evaluating the robustness of the output model as a function of the chosen threshold. fastcore can perform this analysis in a few minutes, whereas for the same problem other algorithms would need hours or days. (Algorithms like GIMME or GIMMEp that require manual curation and assembly of subnetworks, would also fail in this kind of task.) Another example where fast computation is imperative is cross-validation. In the current study (see ‘Results’ section) we ran a random sub-sampling validation procedure 500 times, an operation that took a few minutes with fastcore but that would barely be manageable with other reconstruction algorithms. Other examples where fast computation is important are time-course experiments or experiments involving different patients or conditions [40]. There fastcore could more easily identify differential models over time and/or input conditions.

Compactness is a key concept in various research areas of biology, such as the minimal genome [41], [42]. Notwithstanding, the requirement of model compactness seems to be in disagreement with the observation that biological systems are fairly redundant and this redundancy serves a specific purpose, namely, the fast adaptation to changes in the environment. Alternative pathways that perform similar functions are known to be expressed in different environmental conditions, allowing for instance to metabolize another type of sugar when glucose is not available [43]. At any rate, the pursuit of compactness in metabolic network reconstruction need not be in conflict with the notion of redundancy. Alternative pathways will be included in a reconstructed model as long as ‘redundant’ reactions that are supported by biological evidence are included in the core set.

## Supporting Information

Text S1 **Detailed comparison of the liver models generated with MBA and FASTCORE.** (See main text, Section ‘Reconstruction of a liver model’).
(PDF)
