# Supplementary material for: Fast Reconstruction of Compact Context-Specific Metabolic Network Models
Source: PLoS Comput Biol. 2014 Jan 16;10(1):e1003424. doi: 10.1371/journal.pcbi.1003424 (PMC3894152; doi:10.1371/journal.pcbi.1003424)
Supplement: Text S1 — Detailed comparison of the liver models generated with MBA and FASTCORE. (See main text, Section ‘Reconstruction of a liver model’). (PDF) [file pcbi.1003424.s001.pdf]

Fast reconstruction of compact context-specific metabolic network models

Nikos Vlassis[1], Maria Pires Pacheco[2], Thomas Sauter[2]
1 Luxembourg Centre for Systems Biomedicine, University of Luxembourg, Luxembourg
2 Life Sciences Research Unit, University of Luxembourg, Luxembourg
E-mail: nikos.vlassis@uni.lu

Text S1: Detailed comparison of the liver models generated with MBA and FASTCORE (see main text, Section "Reconstruction of a liver model")

Table S1: Reactions that are exclusively present in the liver model built with FASTCORE

Reaction	Subsystem
'2DR1PP'	'Pyrimidine Catabolism'
'2HBO'	'Propanoate Metabolism'
'2HBt2'	'Transport, Extracellular'
'ABUTt2r'	'Transport, Extracellular'
'ACHVESSEC'	'Transport, Extracellular'
'ACt2m'	'Transport, Mitochondrial'
'ADRNCOAtx'	'Transport, Peroxisomal'
'AKGMALtm'	'Transport, Mitochondrial'
'ALAtN1'	'Transport, Extracellular'
'ARACHDt2'	'Transport, Extracellular'
'ARGNm'	'Urea cycle/amino group metabolism'
'ARTFR204'	'R Group Synthesis'
'BALAVECSEC'	'Transport, Extracellular'
'CATp'	'Miscellaneous'
'COAtm'	'Transport, Mitochondrial'
'CRNtuIR'	'Transport, Extracellular'
'CSNATr'	'Carnitine shuttle'
'CYTD'	'Pyrimidine Catabolism'
'DCMPDA'	'Pyrimidine Catabolism'
'DHPR'	'Tetrahydrobiopterin'
'DM_ethamp(r)'	''
'DNDPt20m'	'Transport, Mitochondrial'
'DNDPt26m'	'Transport, Mitochondrial'
'DNDPt39m'	'Transport, Mitochondrial'
'DOPAtu'	'Transport, Extracellular'
'EX_2hb(e)'	''
'EX_4abut(e)'	''
'EX_ala_B(e)'	''
'EX_amp(e)'	''
'EX_cgly(e)'	''
'EX_dopa(e)'	''
'EX_h(e)'	''

'EX_hxan(e)'	''
'EX_nrpphr(e)'	''
'EX_o2(e)'	''
'EX_xylt(e)'	''
'GLYtm'	'Transport, Mitochondrial'
'GLYtp'	'Transport, Peroxisomal'
'H2Oter'	'Transport, Endoplasmic Reticular'
'H2Otp'	'Transport, Peroxisomal'
'HAS2'	'Hyaluronan Metabolism'
'HDCAter'	'Transport, Endoplasmic Reticular'
'HMGCOAtm'	'Transport, Mitochondrial'
'HYXNt'	'Transport, Extracellular'
'Htr'	'Transport, Endoplasmic Reticular'
'Htx'	'Transport, Peroxisomal'
'NRPPHRtu'	'Transport, Extracellular'
'NTD4'	'Pyrimidine Catabolism'
'O2t'	'Transport, Extracellular'
'O2tp'	'Transport, Peroxisomal'
'OCCOAtm'	'Transport, Mitochondrial'
'PMTCOAtx'	'Transport, Peroxisomal'
'PYRt2m'	'Transport, Mitochondrial'
'RTOT1'	'R Group Synthesis'
'SGPL12r'	'Sphingolipid Metabolism'
'THRGLNexR'	'Transport, Extracellular'
'THRSERNaEx'	'Transport, Extracellular'
'TMNDNCCOAtx'	'Transport, Peroxisomal'
'XYLTt'	'Transport, Extracellular'

Table S2: Reactions that are exclusively present in the liver model built with MBA

Reaction	Subsystem
'5MTHFt'	'Transport, Extracellular'
'5MTHFt2'	'Transport, Extracellular'
'AACOAT'	'Propanoate Metabolism'
'ABTD'	'Pentose and Glucuronate Interconversions'
'ABTti'	'Transport, Extracellular'
'ACACt2m'	'Transport, Mitochondrial'
'ACNAM9PL2'	'Aminosugar Metabolism '
'ACP1_FMN'	'Riboflavin Metabolism'
'ACt2r'	'Transport, Extracellular'
'ADEt'	'Transport, Extracellular'
'ALADGLNexR'	'Transport, Extracellular'
'ALADGLYexR'	'Transport, Extracellular'
'ALAGLYexR'	'Transport, Extracellular'
'ARACHCOAtx'	'Transport, Peroxisomal'
'ARTFR202'	'R Group Synthesis'
'ASCBt'	'Transport, Extracellular'
'ASCBt4'	'Transport, Extracellular'
'ASNtN1'	'Transport, Extracellular'
'BILGLCURte'	'Transport, Extracellular'
'BTNDe'	'Biotin Metabolism'
'CHLtm'	'Transport, Mitochondrial'

'CHOLATEt'	'Transport, Extracellular'
'CHOLt4'	'Transport, Extracellular'
'CHOLtu'	'Transport, Extracellular'
'CLOHtex2'	'Transport, Extracellular'
'CRNt'	'Transport, Extracellular'
'CRNtuNa'	'Transport, Extracellular'
'CRVNCtr'	'Transport, Extracellular'
'CYSGLYexR'	'Transport, Extracellular'
'CYSTSERex'	'Transport, Extracellular'
'CYTDt5'	'Transport, Extracellular'
'DALAt2r'	'Transport, Extracellular'
'DATPtn'	'Transport, Nuclear'
'DHAAt1r'	'Transport, Extracellular'
'DHPR2'	'Tetrahydrobiopterin'
'DM_datp(m)'	''
'DM_datp(n)'	''
'DM_kdn_c'	''
'DNDPt22m'	'Transport, Mitochondrial'
'DNDPt23m'	'Transport, Mitochondrial'
'DNDPt24m'	'Transport, Mitochondrial'
'DNDPt25m'	'Transport, Mitochondrial'
'DNDPt31m'	'Transport, Mitochondrial'
'DNDPt37m'	'Transport, Mitochondrial'
'DNDPt38m'	'Transport, Mitochondrial'
'DNDPt41m'	'Transport, Mitochondrial'
'DNDPt46m'	'Transport, Mitochondrial'
'DNDPt4m'	'Transport, Mitochondrial'
'DNDPt50m'	'Transport, Mitochondrial'
'DNDPt52m'	'Transport, Mitochondrial'
'DNDPt61m'	'Transport, Mitochondrial'
'DNDPt62m'	'Transport, Mitochondrial'
'DNDPt63m'	'Transport, Mitochondrial'
'DNDPt7m'	'Transport, Mitochondrial'
'DRIBt'	'Transport, Extracellular'
'ESTRADIOLtr'	'Transport, Endoplasmic Reticular'
'EX_5mthf(e)'	''
'EX_abt(e)'	''
'EX_ade(e)'	''
'EX_ala_D(e)'	''
'EX_arachd(e)'	''
'EX_biocyt(e)'	''
'EX_btn(e)'	''
'EX_drib(e)'	''
'EX_fru(e)'	''
'EX_gsn(e)'	''
'EX_h2o(e)'	''
'EX_h2o2(e)'	''
'EX_ha_pre1(e)'	''
'EX_hco3(e)'	''
'EX_uri(e)'	''
'FATP3t'	'Transport, Extracellular'
'FATP4t'	'Transport, Extracellular'
'FATP6t'	'Transport, Extracellular'
'FATP8t'	'Transport, Extracellular'
'FATP9t'	'Transport, Extracellular'
'FORt2m'	'Transport, Mitochondrial'

'FRUt4'	'Transport, Extracellular'
'FTHFLm'	'Folate Metabolism'
'FUMTSULtm'	'Transport, Mitochondrial'
'FUMtm'	'Transport, Mitochondrial'
'GALt4'	'Transport, Extracellular'
'GALt4_2'	'Transport, Extracellular'
'GLACter'	'Transport, Endoplasmic Reticular'
'GLCMter'	'Transport, Extracellular'
'GLNLASEer'	'Ascorbate and Aldarate Metabolism'
'GLRASE'	'Ascorbate and Aldarate Metabolism'
'GLUt6'	'Transport, Extracellular'
'GLYCt'	'Transport, Extracellular'
'GSNt5'	'Transport, Extracellular'
'GULLACter'	'Transport, Endoplasmic Reticular'
'H2CO3D2m'	'Miscellaneous'
'H2CO3Dm'	'Miscellaneous'
'HISt4'	'Transport, Extracellular'
'HIStiDF'	'Transport, Extracellular'
'HSD17B1'	'Steroid Metabolism'
'ILEt4'	'Transport, Extracellular'
'INSTt2r'	'Transport, Extracellular'
'INSTt4_2'	'Transport, Extracellular'
'INSt'	'Transport, Extracellular'
'KDNH'	'Aminosugar Metabolism '
'LEUt4'	'Transport, Extracellular'
'L_LACt4r'	'Transport, Extracellular'
'MALSO3tm'	'Transport, Mitochondrial'
'MANt4'	'Transport, Extracellular'
'MI1PP'	'Inositol Phosphate Metabolism'
'MI1PS'	'Inositol Phosphate Metabolism'
'MTHFD2m'	'Folate Metabolism'
'NADHtru'	'Transport, Endoplasmic Reticular'
'NADtru'	'Transport, Endoplasmic Reticular'
'NAIt'	'Transport, Extracellular'
'NCAMUP'	'Transport, Extracellular'
'NH4t3r'	'Transport, Extracellular'
'NKCC2t'	'Transport, Extracellular'
'OCDCEAtr'	'Transport, Extracellular'
'PAIL_HStn'	'Transport, Nuclear'
'PHEt4'	'Transport, Extracellular'
'PIK4n'	'Inositol Phosphate Metabolism'
'PIt7'	'Transport, Extracellular'
'PIt8'	'Transport, Extracellular'
'PIt9'	'Transport, Extracellular'
'PPItr'	'Transport, Endoplasmic Reticular'
'PROt4(2)r'	'Transport, Extracellular'
'PUNP6'	'Purine Catabolism'
'PYNP2r'	'Pyrimidine Catabolism'
'RBFK'	'Riboflavin Metabolism'
'RDH1a'	'Vitamin A Metabolism'
'RETFAt'	'Transport, Extracellular'
'RIBt2'	'Transport, Extracellular'
'SERGLYexR'	'Transport, Extracellular'
'SO4t4_2'	'Transport, Extracellular'
'SO4t4_3'	'Transport, Extracellular'
'THRGLYexR'	'Transport, Extracellular'

'THYOXt2'	'Transport, Extracellular'
'TRPt4'	'Transport, Extracellular'
'UREAt'	'Transport, Extracellular'
'URIt4'	'Transport, Extracellular'
'UROLACer'	'Ascorbate and Aldarate Metabolism'
'VITD3t'	'Transport, Extracellular'

Figures S1-S18: Examples where MBA and FASTCORE selected different non-core reactions to add to the core set. Reactions exclusively present in the liver models built with MBA and FASTCORE are depicted in pink and blue, respectively. Reactions present in both models are given in gray. Core reactions are colored in green.

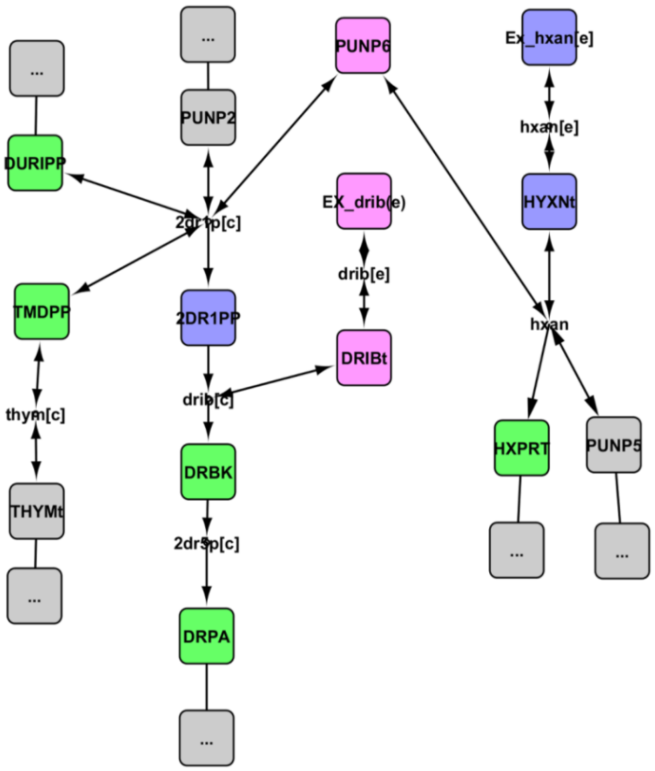

Figure S1: MBA and FASTCORE select two different exchange reactions to connect this part of the network to the environment. FASTCORE considers this part of the network as two independent subnetworks (left and right of the PUNP6 reaction), whereas MBA divides the left subnetwork in upstream and downstream of the reaction 2DR1PP.

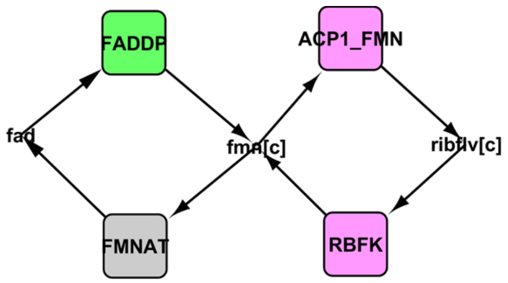

Figure S2: In this example, MBA adds an unnecessary loop to the network.

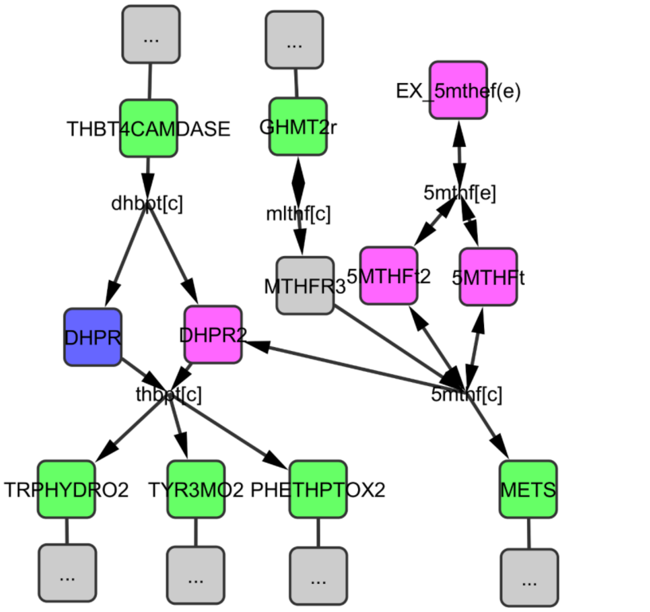

Figure S3: MBA connects the system to the environment via a transporter, whereas FASTCORE adds an isozyme (DHPR instead of DHPR2) which allowed reducing the number of necessary non-core reactions (here 5MthFt2, 5MTHFt and EX_5methef[e]).

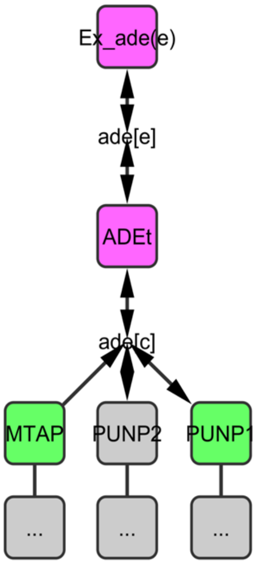

Figure S4: MBA connects the network to the environment, whereas FASTCORE does not.

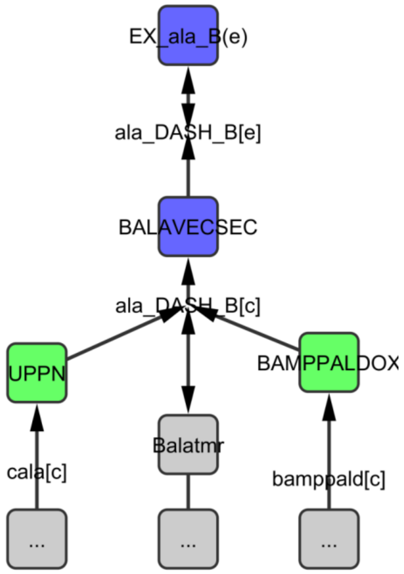

Figure S5: FASTCORE connects the network to the environment, whereas MBA does not.

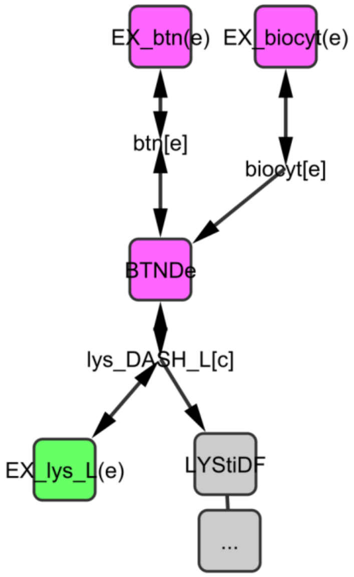

Figure S6: MBA adds three reactions in order to connect the network to the environment, although the network is already connected via EX_lys_L.

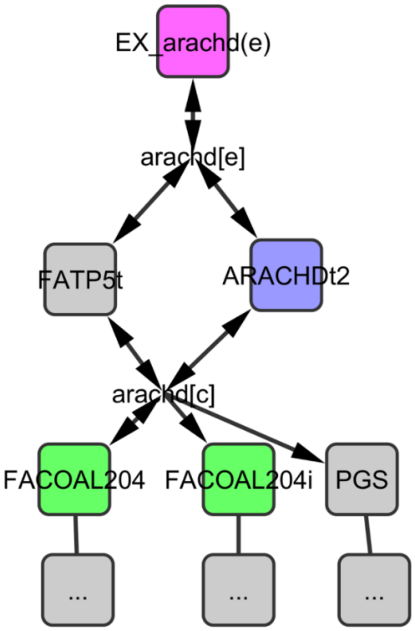

Figure S7: MBA connects the network to the environment, whereas FASTCORE adds a transporter.

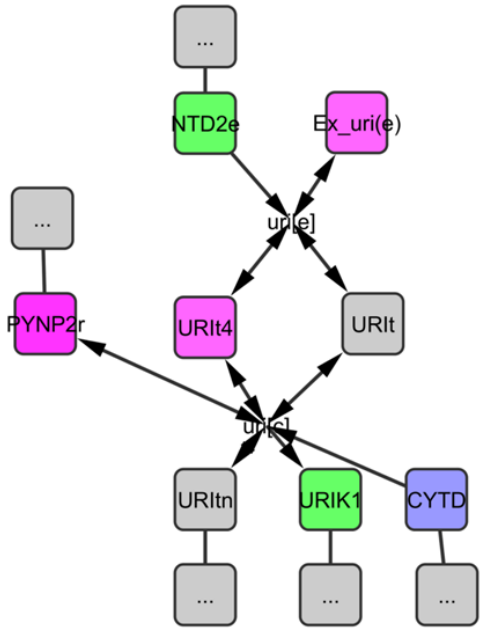

Figure S8: MBA connects the network to the environment and keeps an unnecessary transporter.

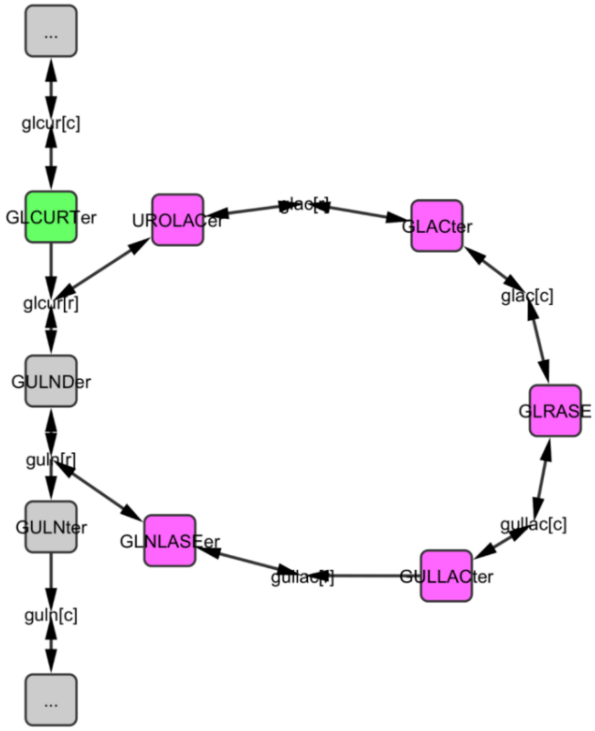

Figure S9: MBA keeps an unnecessary loop.

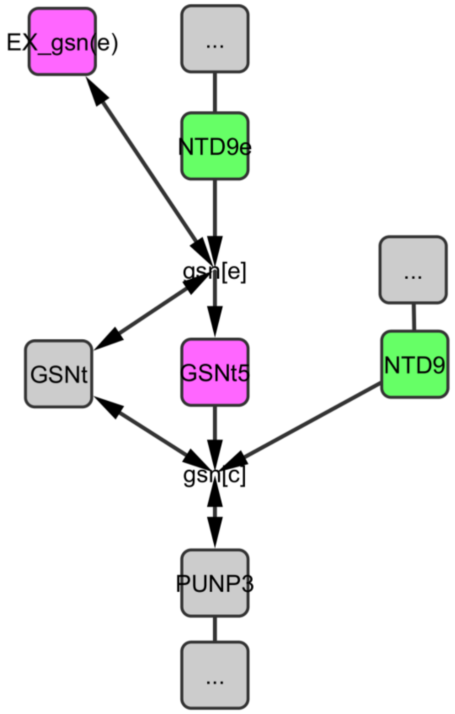

Figure S10: MBA connects the network to the environment and keeps an unnecessary transporter.

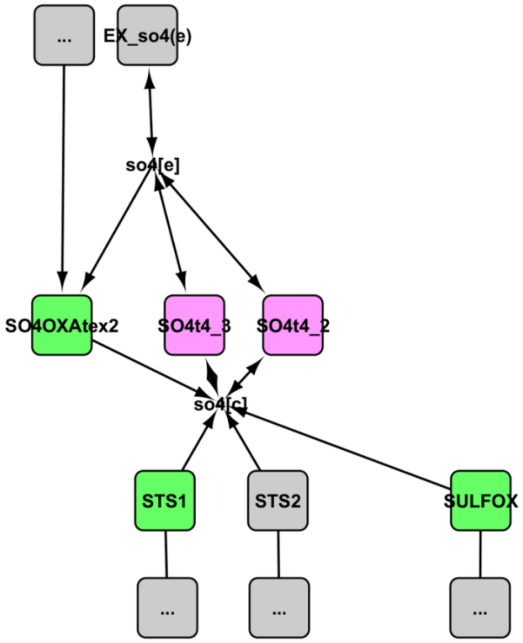

Figure S11: MBA keeps the three alternative pathways.

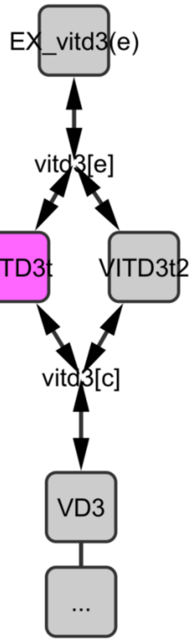

Figure S12: MBA keeps two alternative pathways.

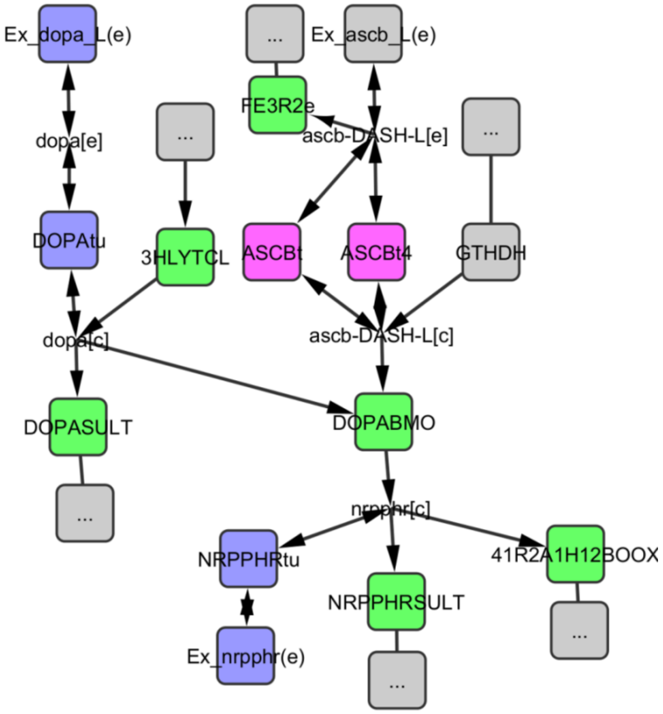

Figure S13: FASTCORE adds exchange reactions, whereas MBA keeps two transporters.

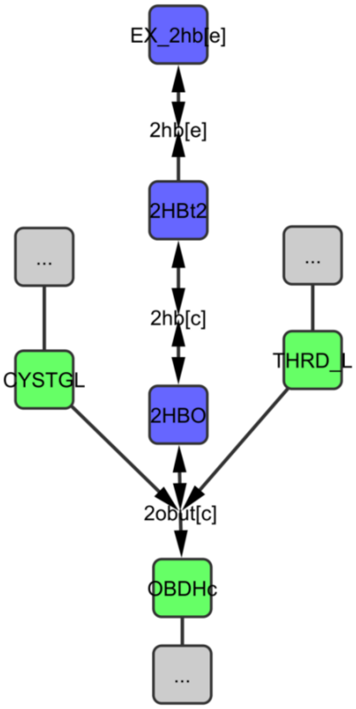

Figure S14: FASTCORE adds exchange reactions.

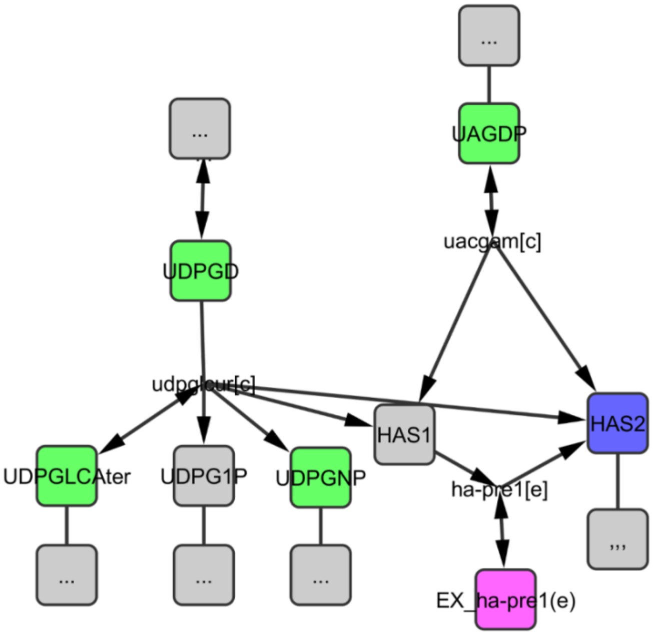

Figure S15: MBA selects to keep an exchange reaction, whereas FASTCORE adds a reaction (HAS2).

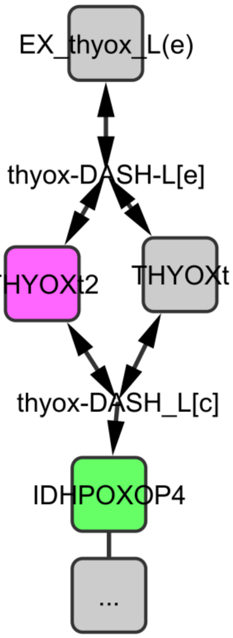

Figure S16: MBA keeps the two transporters, whereas one would have been sufficient.

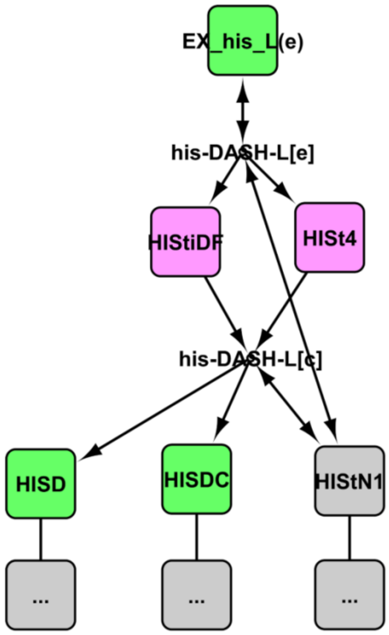

Figure S17: MBA keeps the three transporters, whereas one would have been sufficient.

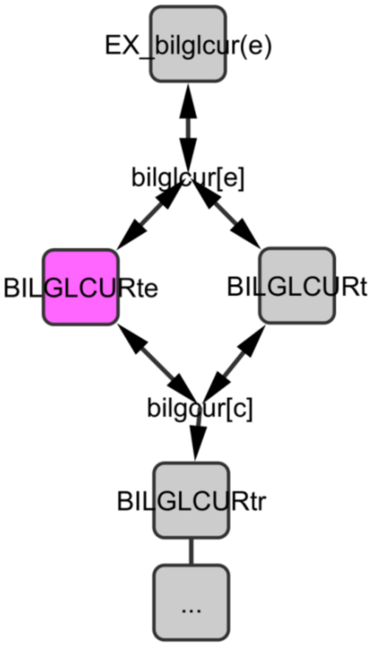

Figure S18: MBA algorithm keeps the two transporters whereas one would have been sufficient.
